# Estimates of regeneration potential in the Pannonian sand region help prioritize ecological restoration interventions

**DOI:** 10.1038/s42003-022-04047-8

**Published:** 2022-10-27

**Authors:** Edina Csákvári, Zsolt Molnár, Melinda Halassy

**Affiliations:** 1 ELKH Centre for Ecological Research, Institute of Ecology and Botany, Restoration Ecology Research Group, Alkotmány u. 2-4, 2163 Vácrátót, Hungary; 2 ELKH Centre for Ecological Research, Institute of Ecology and Botany, Traditional Ecological Knowledge Research Group, Alkotmány u. 2-4, 2163 Vácrátót, Hungary

**Keywords:** Restoration ecology, Grassland ecology

## Abstract

Restoration prioritization helps determine optimal restoration interventions in national and regional spatial planning to create sustainable landscapes and maintain biodiversity. Here we investigate different forest-steppe vegetation types in the Pannonian sand region to provide restoration recommendations for conservation management, policy and research. We create spatial trajectories based on local, neighbouring and old-field regeneration capacity estimates of the Hungarian Habitat Mapping Database, compare the trajectories between different mesoregions and determine which environmental predictors possibly influence them at the mesoregion level using a random forest model. The trajectories indicate which types of passive or active restoration intervention are needed, including increasing connectivity, controlling invasive species, or introducing native species. Better restoration results can be achieve in the vicinity of larger (semi-)natural areas, but the specific site conditions must also be taken into account during prioritization. We also propose large-scale grassland restoration on abandoned agricultural fields instead of industrial forest plantations and afforestation with non-native species.

## Abstract (web summary)

A case study of forest-steppe vegetation types in the Pannonian sand region of Hungary reveals avenues for ecological restoration via estimated regeneration potential.

## Introduction

Human activities transform the natural environment and cause a decline in biodiversity and ecosystem services. According to a recent global assessment report by the Intergovernmental Science-Policy Platform on Biodiversity and Ecosystem Services (IPBES) the main threats to biodiversity are land degradation and habitat fragmentation^1^. Expansion of intensive agriculture, loss of traditional land use, improper forest management practices, and invasion of alien species are the main drivers of land degradation^1^. The United Nations (UN) has recently declared the Decade on Ecosystem Restoration for the 2021–2030 period^2^, which provides an unparalleled opportunity for science–policy discussion to focus on future restoration^3^. The aim is to restore 350 million hectares of degraded terrestrial and aquatic ecosystems worldwide by 2030, that is projected to cost US$1 trillion, representing 0.1% of the global economic output between 2020 and 2030. Restoration is a valuable investment for both nature and society, creating US$9 trillion in ecosystem services and contributing to poverty alleviation^2^.

Prioritization of ecological restoration interventions is important in developing the strategic framework needed to ensure the future health and stability of ecosystems^4^, while the science-based prioritization of areas to be restored in national and regional planning contributes to the creation of a sustainable landscape that maintains biodiversity and ecosystem services^1^. Recent research suggests that restoring 15% of converted land in global priority areas could prevent 60% of expected extinctions and mitigate climate change^5^. Of the major ecosystem types, wetlands and forests have proven to be the most important in the latter. But if the goal is both restoration and minimizing monetary costs, then arid ecosystems and grasslands have the highest importance^5,6^. Further cost reductions can be achieved by optimizing the allocation and restoration methods during spatial planning^5,7–9^.

In the European context, steppe habitats are of community importance in the drier part of the aridity gradient. These habitat types are part of the Eurasian forest-steppe region and are characterized by a high level of species and functional diversity^10^. In addition to their ecological value, grasslands have extraordinary social and economic significance, as they have been used as extensive pastures and meadows for centuries^11,12^. Unfortunately, the remaining (semi-)natural habitats are at risk due to large-scale land degradation and habitat fragmentation caused by human activities^13^. A big challenge for policymakers, researchers, and practitioners is to jointly protect and restore the remaining (semi-)natural steppe areas. This can be facilitated by setting restoration priorities and determining optimal restoration interventions at the regional and national levels. In data-driven restoration planning, both ecological and economic factors should be considered to choose the optimal restoration action^5,9,14,15^. Passive restoration relies on natural processes without external intervention, preferred on sites with low abiotic stress and moderate disturbance, and in landscapes that are less affected by humans. It is an economically viable choice for producing ecological and social benefits and for achieving cost-effective large-scale restoration, but it also requires knowledge about environmental factors that could limit effectiveness and success^8,16,17^. Active restoration should be preferred for enhancing biotic and abiotic site conditions, but the intervention’s costs are going to be higher than passive restoration^8^.

During the prioritization process, we suggest the most ecologically and economically appropriate active and passive restoration practices based on the regeneration capacity of local habitats, neighboring vegetation patches, and old fields. We focused on the analysis of spatial regeneration trajectories for different forest-steppe vegetation types (open and closed sand grasslands, poplar-juniper sand dune forests, and thickets), and their role in assessing landscape-scale recovery related to the future natural dynamics of habitats under human pressure^18^. Trajectory analysis is a spatially driven method that allows for the qualitative representation of spatial and temporal data^19–25^. Here we aimed to provide ecological restoration recommendations based on spatial regeneration trajectories for policy, conservation management, and research at the mesoregion level. Our questions were: (i) What are the spatial regeneration trajectories of Pannonian sandy habitat types in the studied mesoregions? (ii) Which biotic and abiotic environmental factors determine the spatial regeneration trajectories? (iii) What are the most efficient restoration methods based on local regeneration capacity and spatial regeneration trajectories? (iv) How can restoration efforts be prioritized?

Habitat-specific spatial trajectory analysis revealed important regeneration differences between locations and regions. The trajectory shapes were used to define factors limiting recovery and were linked to suggested types of passive or active restoration intervention. Our results highlight that better restoration results can be achieved near existing larger (semi-)natural areas where limiting disturbances are low, but when determining the restoration priorities, the altered site conditions, the landscape context, and possible future changes due to climate change should also be scrutinized. Large-scale land abandonment offers potential for regeneration of biodiversity and climate mitigation, and non-forested ecosystems should be considered as an alternative to afforestation, especially where predicted climates are less suitable for native tree species. We hope that the recommended prioritized actions will help to achieve important conservation goals in practice. In addition, the resulting strategies can be applied with minor adjustments to many other vegetation types in the Eurasian forest-steppe region.

## Results

### Spatial regeneration trajectories in the studied mesoregions

**Fig. 1:**
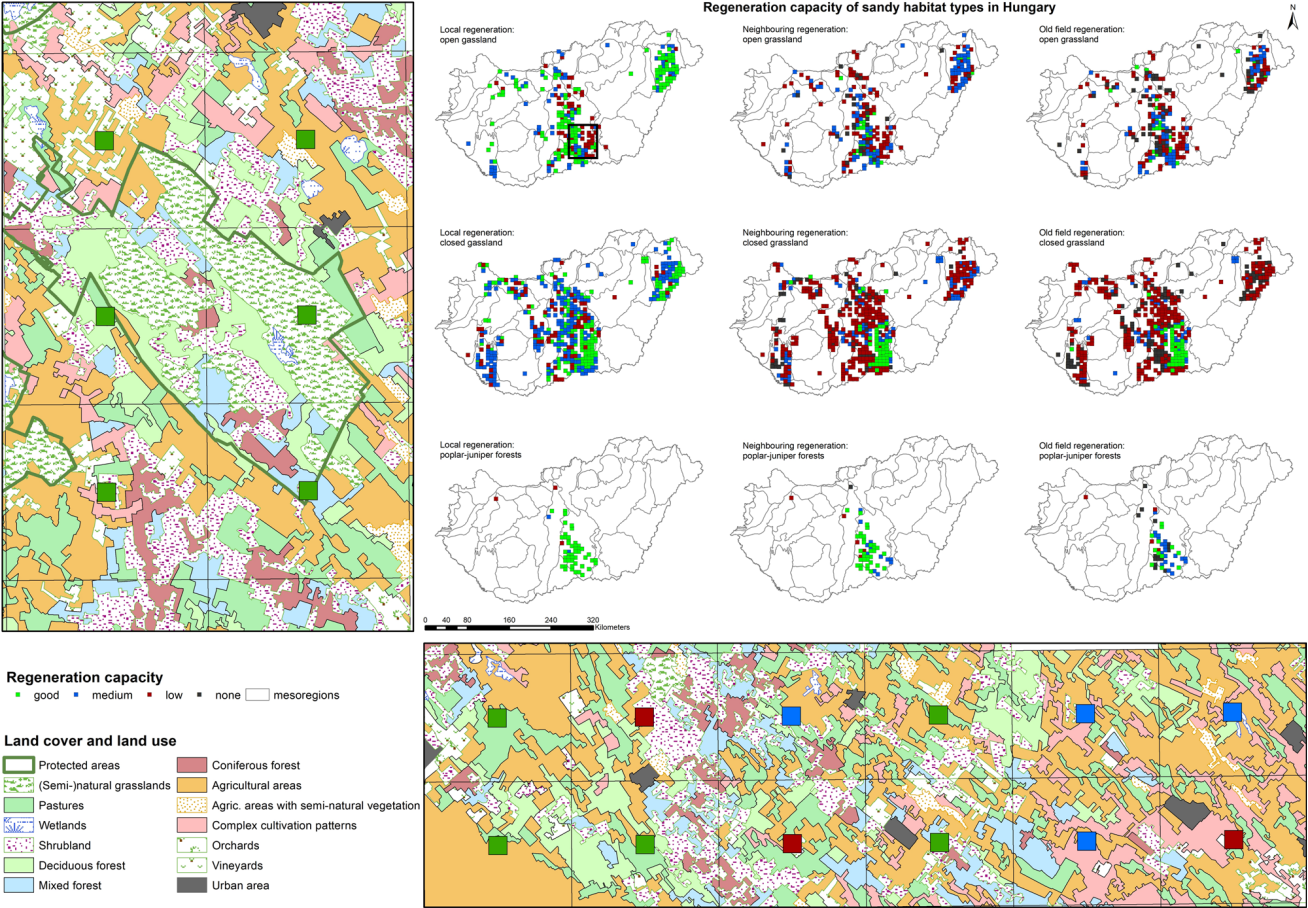
Local, neighboring, and old-field regeneration capacity of sandy habitat types in Hungary. Countrywide distribution of local, neighboring, and old-field regeneration capacity of open sand steppes (*n* = 271), closed sand steppes (*n* = 498), and poplar-juniper sand dune forests and thickets (*n* = 52) and two sample areas showing typical land use (based on Hungarian Habitat Mapping Database^68–70^ and European CORINE Land Cover 2006 database^72^) in the sandy regions of Hungary.

**Fig. 2:**
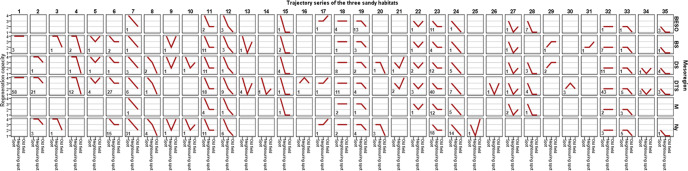
Spatial regeneration trajectories of the three habitat types in the studied mesoregions. Sandy habitat types in the Hungarian mesoregions: open sand grasslands *n* = 223, closed sand grasslands *n* = 363, and poplar-juniper sand dune forests and thickets *n* = 51; x-axis: three different locations (spot, neighboring spot, old-field); y-axis: regeneration capacity (good (4), moderate (3), low (2), none (1)). The abbreviations for each mesoregion: BESO Belső-Somogy, BS Bácskai-síkvidék, DS Dunamenti-síkság, DTS Duna-Tisza közi síkság, M Mezőföld, Ny Nyírség. The numbers in the boxes represent the exact values of each spatial regeneration trajectory.

According to the local, neighboring, and old-field regeneration capacity of the sandy habitats (Fig. 1), the spatial regeneration trajectories were classified into four major types. There is a wide variation at the mesoregion level (Fig. 2): (i) A flat trajectory means that the regeneration capacity is the same locally, on neighboring vegetation patches, as well as on old fields. (ii) In most cases, the trajectory declines from local habitats towards old fields. (iii) A “V-shape” trajectory means old-field regeneration is better than regeneration on neighboring vegetation patches. Finally, (iv) the increasing trajectory means regeneration capacity is better in neighboring areas and/or old fields than locally. The prevalence and exact values of trajectories in each habitat type and mesoregion are given in Supplementary Data 1.

In the case of open sand grasslands, we found significant differences between the trajectories of the mesoregions Nyírség and Dunamenti-síkság, as well as Nyírség and Duna-Tisza közi síkság at the *p* < 0.05 significance level. Spatial autocorrelation was low: Moran’s I = 0.13; *p* < 0.01 (Fig. S1). At the closed sand grasslands, the mesoregion pairs whose trajectories showed significant differences were Belső-Somogy and Duna-Tisza közi síkság; Belső-Somogy and Nyírség; Duna-Tisza közi síkság, and Dunamenti-síkság; Duna-Tisza közi síkság and Mezőföld (*p* < 0.05). Spatial autocorrelation was also low: Moran’s I = 0.22; *p* < 0.0001 (Fig. S2). At the juniper-poplar stands, significant differences were found between the trajectories of Bácskai-síkvidék and Dunamenti-síkság; Dunamenti-síkság and Duna-Tisza közi síkság (*p* < 0.05). There was no significant spatial autocorrelation (Moran’s I = 0.05; *p* = 0.29; Fig. S3). (The exact values of one-way ANOVA analysis and Tukey’s post hoc significance test between mesoregions is given in Tables S1–S6.)

### Environmental predictors that determine spatial regeneration trajectories

In the case of open sand grasslands (*n* = 223; classification accuracy 0.65; error rate 39%), the main environmental predictor determining the spatial regeneration trajectories was temperature seasonality (importance 16.4%), followed by topography (15.6%), groundwater level (13.4%) and naturalness (13.4%), and to a lesser extent by other environmental factors. The spatial regeneration trajectories of the closed sand grasslands (*n* = 363; classification accuracy 0.81; error rate 22.9%) were mainly determined by topography (14.9%), the extent of local habitats (14.3%), and groundwater level (12.3%). In juniper-poplar stands (*n* = 51; classification accuracy 0.70; error rate 24.4%), the main determining predictor was the presence of forest land cover (47.98%), followed by naturalness (23.7%) and seasonality of precipitation (22.9%). The mean decrease in the accuracy of environmental predictors is demonstrated in Fig. S4.

### Restoration prioritization based on local regeneration capacity

**Fig. 3:**
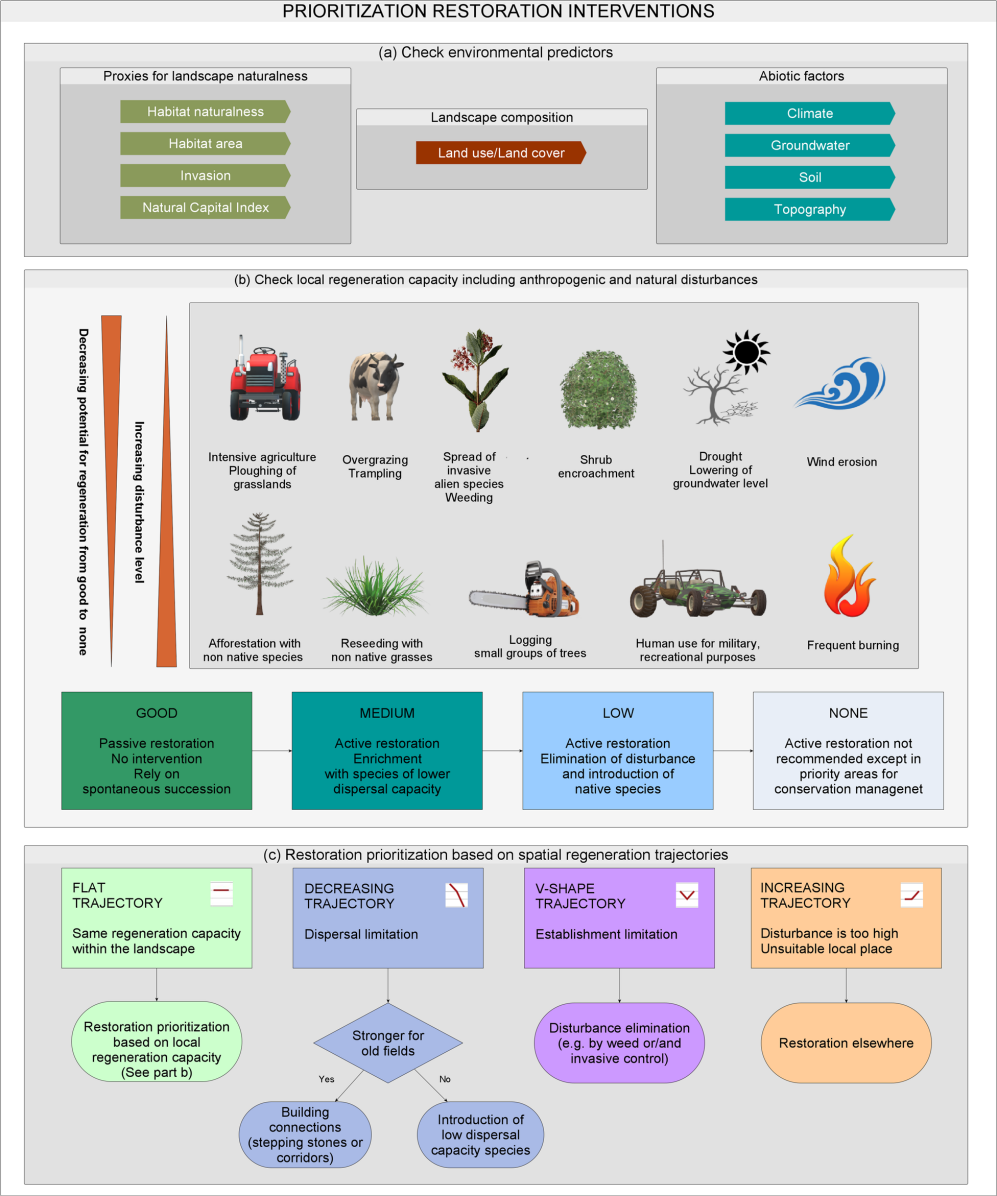
Conceptual diagram of prioritizing restoration interventions in the sandy habitat types. **a** Studied environmental predictors, **b** restoration prioritization based on the local regeneration capacity including anthropogenic and natural disturbances, and **c** restoration prioritization based on the spatial regeneration trajectories. (The icons in Fig. 3b were created using Microsoft Paint 3D software.).

When only the local regeneration capacity is studied, the definitions related to the four-grade scale provide guidance for the type of restoration interventions required (Fig. 3b):

By definition, regeneration capacity is considered good if there are adequate sources of species and suitable sites for regeneration within the landscape, which is likely to provide similar (semi-)natural habitats as a result of regeneration. This means that when prioritizing action for restoration, all areas with good regeneration capacity can be left to passive restoration, namely spontaneous succession.

In the case of medium regeneration capacity, the regeneration process is either too slow or does not reach the natural state by itself (e.g., specialist species are lacking and disturbance level is medium). This calls for active interventions to overcome the weed-dominated stages and accelerate succession. Since the process itself can start, it should generally need only enrichment with species of lower dispersal capacity.

If the regeneration capacity is low, the process may start, and a few dominant species can colonize, but the process stops in a stage dominated by weeds due to the lack of propagules or the rapid spread of weeds and/or invasive species. In such cases, more active intervention is needed. Depending on the cause of low regeneration capacity, elimination of disturbances (e.g., weed/invasive control depending on the type of disturbance) and introduction of native grassland species (e.g., by sowing of high diversity seed mixtures or hay transfer) will be necessary to achieve a better status.

Regeneration is considered impossible if there is a lack of suitable spaces for colonization. It follows that restoration in such landscapes is not recommended due to the high costs of the required change in habitat types, except for the highest nature conservation priorities.

### Restoration prioritization based on spatial regeneration trajectories

When considering the three locations of regeneration capacity together as a trajectory, we get a more nuanced picture (Fig. 3c). If the spatial regeneration trajectory yields a straight line (Fig. 2 series 1, 18, 32), that means that the limiting factors and potential for regeneration is the same everywhere within the landscape and similar restoration measures are required: passive restoration if the regeneration capacity is good, enrichment in case of medium regeneration capacity, and elimination of disturbances and introduction of native species for low regeneration capacity areas.

If the trajectory is decreasing, it is a sign of dispersal limitation (Fig. 2 series 7, 8, 12, 24). The steeper the decline, the stronger this constraint. If the dispersal limitation is visible only for old fields, but not for neighboring vegetation types (Fig. 2 series 2, 3, 4, 19, 20, 33), it is a sign that we can rely on shorter-range dispersal during restoration by building connections (stepping stones or corridors) between the remnant habitat patches and the farther old fields. If the regeneration capacity is similar in neighboring vegetation patches and on old fields, it is a sign of strong dispersal limitation. Consequently, building connections is not enough, enrichment (Fig. 2 series 6) or direct introduction of species (Fig. 2 series 11, 23) is required. In three series (Fig. 2 series 15, 28, 35), only local regeneration is possible, that calls for the strict protection of still existing habitat remnants, even if degraded, since without it, the habitat would disappear.

In certain trajectories (Fig. 2 series 5, 9, 10, 13, 14, 21, 22, 25, 26, 34) old-field regeneration is better than regeneration on neighboring vegetation patches. This is a sign that factors other than dispersal limit the regeneration in the neighboring areas that are likely to be associated with the existing disturbances (the main difference compared to old fields). In such cases, the elimination of disturbance (e.g., control of existing vegetation, land use change) might be necessary to help the recovery of the habitat.

Finally, in a few series (Fig. 2 series 16, 17, 29, 30, 31), regeneration capacity is better in neighboring vegetation types and/or old fields than locally. This indicates that the present location is less suitable for the habitat than others, e.g., many closed grasslands dry out locally due to the decrease in the groundwater level, but their regeneration can occur in place of drying wetlands. In such cases, restoration efforts should be focused on the sites with the best regeneration capacity.

### Prioritized restoration interventions on the mesoregion level

In *Belső-Somogy*, most trajectories showed a decline only for old fields (series 19, *n* = 13). Here we recommend active restoration by building stepping stones or corridors to assist longer-range dispersal. Where the regeneration capacity was equally low in neighboring vegetation types as on old fields (23, *n* = 11), shrub control is proposed after the direct introduction of species (e.g., sowing of high diversity seed mixtures or hay transfer). In *Bácskai-síkvidék* and in *Mezőföld* the most common trajectory series was decreasing, indicating good or moderate regeneration locally, but worse regeneration elsewhere (series 11, *n* = 11; series 23, *n* = 12, respectively), therefore the introduction of species is the most suggested restoration method. In the case of *Dunamenti-síkság*, the most common trajectory series were similarly decreasing (23, *n* = 12; 11, *n* = 11), but a flat trajectory was also common (32, *n* = 11). The latter means that the regeneration capacity is low everywhere in the landscape, so in addition to the strict protection of any remnant habitats, species introduction and elimination of disturbances are also required in this mesoregion. In *Nyírség* the most common trajectories were continuously decreasing (7, *n* = 31; 24, *n* = 14) or regeneration capacity was similar in neighboring vegetation types and on old fields (6, *n* = 15; 23, *n* = 18; 11, *n* = 11). Depending on the goodness of regeneration, we suggest either enrichment (moderate regeneration) or disturbance elimination (e.g., weed and/or invasive control), plus native grassland species introduction (lower regeneration capacity). The *Duna-Tisza közi síkság* is the largest mesoregion with the most varied trajectories. Here the flat trajectories were common; therefore, we recommend similar restoration interventions on spot, on neighboring spots, and on old fields: passive restoration (1, *n* = 58), enrichment (18, *n* = 11), and disturbance elimination with species introduction (32, *n* = 43). Next to the straight lines, decreasing trajectories were also prominent. We suggest building connections between the remnant habitat patches and the further old fields (2, *n* = 21), enrichment (6, *n* = 27), and direct introduction of species (11, *n* = 18; 23, *n* = 40) on neighboring vegetation patches and on old fields.

## Discussion

Habitat-specific spatial trajectory analysis revealed important regeneration differences between locations and regions. We found that in most cases, the trajectory declined from local habitats toward old fields. This means that the potential for regeneration is the best on spot, followed by directly adjacent vegetation patches, and the chance for regeneration is the lowest in abandoned fields depending on the level of landscape transformation by humans.

As a result of landscape changes, much of the original vegetation was destroyed in the sandy mesoregions^26–28^. In landscapes that are least affected by humans and habitats might have good regeneration capacity everywhere (flat trajectory) due to the remnant (semi-)natural vegetation (in our case, mainly in *Duna-Tisza közi síkság*). In landscapes where natural remnants are still available (e.g., abandoned pastureland), the spatial regeneration capacity declines only slightly with increasing distance (e.g., *Belső-Somogy*). In landscapes largely transformed by arable cultivation, a sharper decline in spatial regeneration trajectories is expected (like in *Bácskai-síkvidék* and *Mezőföld*). If the current land use or the spread of non-native invasive species impedes propagule dispersal and spontaneous vegetation development, spatial regeneration trajectories decline the most sharply (e.g., *Nyírség* and *Dunamenti-síkság*).

The basic drivers of the decline are the same in Hungary and other Central and Eastern European countries: intensive agriculture and forestry, but there are differences in the regional patterns of agricultural land use and the resulting degradation^29,30^. Another typical change is that extensively used lands were abandoned^31,32^, and former pasture and species-rich hay meadows are threatened today by invasive alien species^33,34^. The major difference between the studied regions lies in the divergence in the type of abandoned cultivation: arable fields in the Great Hungarian Plain, as opposed to pastures in the Transdanubian Hills. This discrepancy is coupled with the differences in the main drivers that hinder recovery, such as overgrazing and trampling in the former^35^, and a strong shrub encroachment due to the lack of grazing in the latter mesoregion^36^. Regeneration of old fields is also hampered by the spread of invasive species in both regions, but the species are partially different, mainly *Robinia pseudoacacia* and *Asclepias syriaca* in the Great Hungarian Plain and *Prunus serotina* and *Phytolacca americana* in the Transdanubian Hills^37^.

Spatial trajectory analysis is often impossible due to scarce information on the regeneration capacity locally, on neighboring spots, and on old fields. In such cases, we can rely on environmental information to provide guidance on regeneration estimation. The area and naturalness of habitats correspond well to the spatial regeneration trajectory of sandy vegetation at the mesoregion level and also to the good regeneration capacity for all three habitat types studied at the country level^38^. This essentially means that in extensively used landscapes with a high amount of (semi-)natural habitats, regeneration capacity can be assumed to be good (Fig. 1).

Abiotic conditions necessarily govern the sustainability of habitats^39^. Altitude and groundwater level are good predictors of regeneration for open and closed steppes, the former occupying dune tops far from the groundwater, the latter preferring inter-dune depressions, and higher groundwater levels with more humus-rich soils. The sand content of the soil is less important in determining spatial regeneration trajectories than the regeneration of individual habitat types at the country level^38^. This suggests that water availability principally determines habitat distribution and outlines well where the restoration of open and closed grasslands is suggested. Open sand grasslands can expand in sandy areas at the expense of the other forest-steppe components primarily due to groundwater drainage that results in unsuitable conditions for other habitats. As groundwater levels fall, the closed sand grassland retreats from drier areas, but can spread to former wet grasslands^38,40^. These processes should be also considered when planning restoration under changing climatic conditions^41^.

Land use and land cover also control regeneration capacity. E.g., the presence of forest land cover indicates a better potential for regeneration of woody habitat types. The presence of agricultural land is a good indicator of the regeneration of sandy habitats, as cropland abandonment is common in low-productivity areas and offers space for the recovery of semi-natural vegetation^42^. However, abandoned croplands are often used for plantation forests^25^, which hinders the regeneration potential of grasslands due to the presence of invasive species^43^. Invasion is a major biotic threat to grassland regeneration, and this threat is stronger in less stressful environments^33^, like in our case, closed steppe vegetation. Invasive species threaten not only spontaneous succession, but also the active restoration of these areas^44,45^.

Regeneration capacity is a good indicator of the potential for passive restoration based on spontaneous processes^16,46^, which is one of the most commonly used restoration techniques^47–50^. It is cost-effective; the only costs are extensive mowing or grazing, which help propagate target species and suppress weeds^7,48^. Passive restoration is a viable alternative in areas where the (semi-)natural vegetation is still existing and can provide a sufficient source of target propagules, this can be indicated also by knowledge of regeneration trajectories or high naturalness of the landscape and environmental conditions that correspond to the target habitats. One of the main tasks for conservationists and decision-makers would be to protect the remnant (semi-)natural areas in order to preserve the potential of the landscapes for recovery.

If the landscape is fragmented locally that is also indicated by medium or low potential for recovery and declining regeneration trajectories, or a limited extent of natural areas, passive restoration can be slow or unpredictable, so in that case, active restoration interventions are required. Regeneration is often hindered by the lack of propagules of native species due to missing nearby propagule sources or limited dispersal ranges^51,52^. If connectivity is low between the remnant habitat patches and the restorable sites, facilitating the migration of native species should be a high nature conservation priority. Similar to the findings of other studies, we suggest improving landscape connectivity by building stepping stones or green corridors^53^. In certain cases, the limitation exists for only a few species, in such cases, enrichment with species of low dispersal ability is suggested.

In the case of strong dispersal limitation, especially considering the high risk of non-native invasion, building connections is not sufficient, but the direct introduction of target species is required. Prach et al.^49^ and Kirmer et al.^54^ found that the use of regional seed mixtures rich in native species and functional groups is more successful than using low diversity or commercial mixtures. Other studies suggest that low diversity seed mixtures can also lead to satisfactory results depending on other constraints (e.g., costs and availability of seeds), although might require further management^47,55^.

For low regeneration capacity areas where the anthropogenic or natural disturbance is high, besides sowing, weed, and/or shrub control, and in certain cases, land-use change is required as well. If the local habitat is large enough, and the aim is to increase species richness, we also suggest hay transfer from local donor sites for consideration, which not only helps introduce target species, but also suppresses weeds^56^. If there is no potential for regeneration due to the lack of space for colonization, the restoration costs would probably be too high; moreover, the success of restoration would be unpredictable and unsustainable in the long run, thus we do not recommend any restoration action except in areas with high conservation priorities.

In addition to the ecological value, optimizing the costs of restoration projects is also important for practitioners to allocate restoration resources and prioritize efforts^9,14,15^. With the help of regeneration trajectories—or based on the environmental factors that correlate well with regeneration capacity—we can optimize the spatial allocation of restorative interventions. Regeneration of sandy habitats in old fields is considerably good, so these areas need to be prioritized to increase the restored area of grasslands instead of a newly created forest of often non-native origin^5,6,57,58^. Old fields could provide an opportunity for passive or active ecological restoration;^49,59^ but the previous negatives still highlight the importance of taking into account the past and ongoing land use and the typical disturbances that limit regeneration in the landscape when planning restoration. Priority should be given to areas where remnant patches of natural vegetation are still present, and we can better rely on passive processes^8,17^. Restoring disturbed areas in their buffer zones and linking the remnant patches by creating stepping stones or corridors can minimize the costs and maximize the benefits of restoration. On the contrary, restoration efforts in highly disturbed and especially highly invaded areas—e.g., in the presence of non-native plantations within the landscape, since these are hot spots for invasive species^43^—might increase the restoration costs and threaten the sustainability of restored areas^5,6,57^.

The analysis of spatial regeneration trajectories offers an opportunity for better restoration prioritization. Although the spatial regeneration trajectories were investigated for sandy habitats, this approach could easily be adapted to other habitat types relying on different environmental predictors of regeneration capacity. Databases of habitats and landscape ecology (such as MÉTA), which often contain environmental proxies suitable for regeneration estimation, provide an excellent basis for this. Our research emphasizes the importance of strategic restoration planning at both national and regional scales to maximize ecological benefits and minimize socio-economic costs. In Hungary, the first steps have already begun with the planning of the Hungarian green infrastructure^60^. Based on our results, regeneration trajectories can be integrated into the spatial planning of green infrastructure development and help develop the necessary implementation practices and related cost estimates. In the future, we plan to develop a decision support system for this purpose.

We hope that our spatial trajectory analysis provides new information on optimal restoration methods for practitioners and decision-makers to conserve and restore valuable habitats and landscapes throughout the Eurasian forest-steppe region and motivates researchers to conduct similar research in other parts of the world.

## Methods

### Study area

Hungary is located in the Carpathian Basin, in Central Europe. Its total area is 93.033 km^2^. The climate is continental, which is somewhat affected by the Atlantic and Mediterranean air masses, and modified by the topography of the basin. The long-term average annual precipitation sum is distributed between 453 and 879 mm within the country. The mean annual temperature is about 10 °C^61^.

**Fig. 4:**
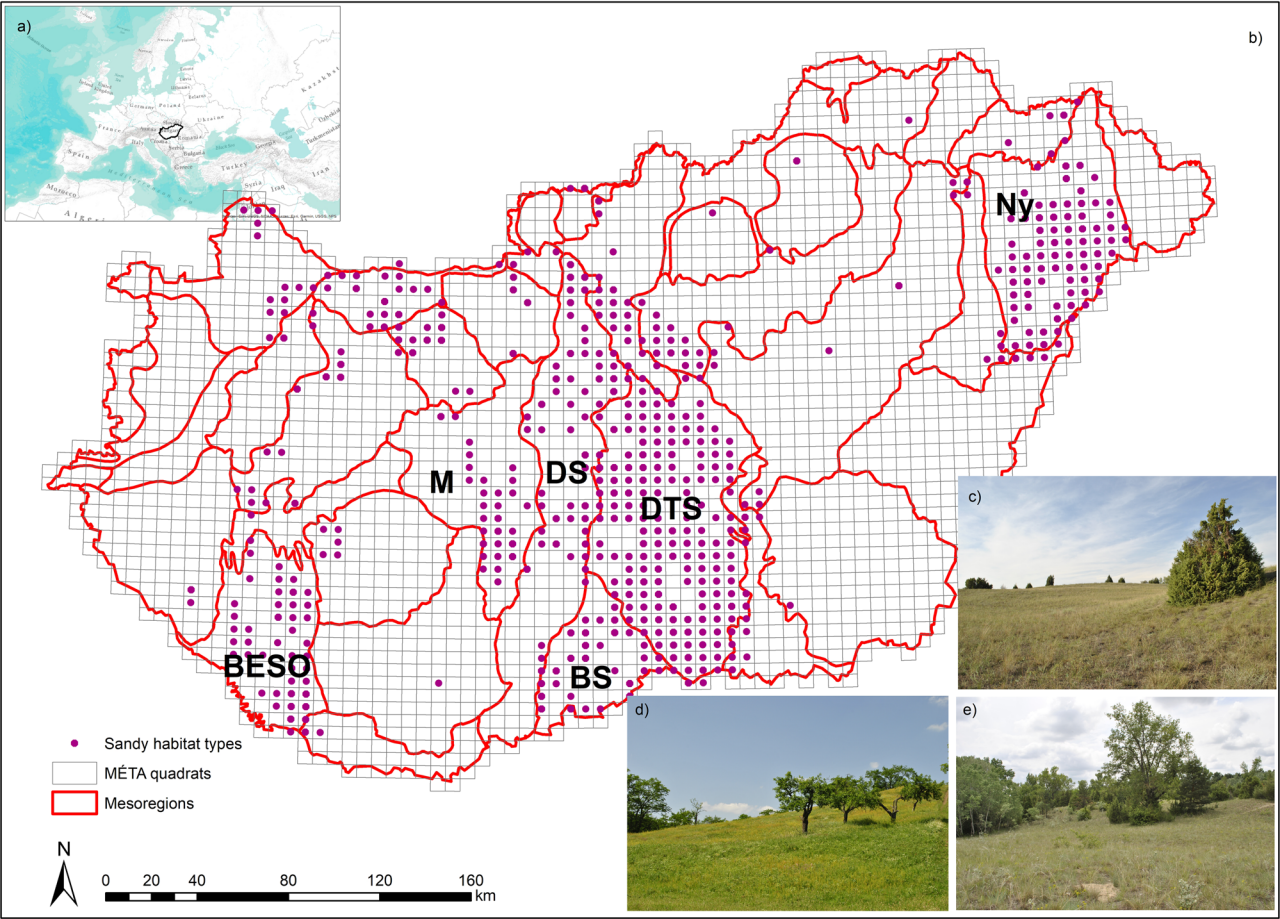
Map of the study area. **a** Hungary’s location in Europe; **b** the studied mesoregions, the quadrat level grid of the Hungarian Habitat Mapping Database (MÉTA^68–70^), and the occurrence of sandy habitats; **c** open sand steppe; **d** closed sand steppes; **e** poplar-juniper sand dune forests and thickets. Abbreviations for each mesoregion: BESO Belső-Somogy, BS Bácskai-síkvidék, DS Dunamenti-síkság, DTS Duna-Tisza közi síkság, M Mezőföld, Ny Nyírség. Photos by Edina Csákvári.

We studied the sandy mesoregions of the forest-steppe biome in the Pannonian biogeographic region in the Hungarian Plain (namely *Duna-Tisza közi síkság*, *Dunamenti-síkság*, *Bácskai-síkvidék*, *Mezőföld*, and *Nyírség)* and in the Transdanubian Hills (namely *Belső-Somogy*) (Fig. 4). The driest areas are in the Hungarian Plain, filled with alluvial sandy deposits originating mostly from the Carpathian Mountains. During the Pleistocene and Holocene, sand and loess layers were rearranged by the wind^62^. In the central and southern parts of the Hungarian Plain, the main soil type is calcareous sandy soil with a humus content below 3%^59^. In the northeast, the main soil type is acidic sandy soil, also low in organic carbon^63^. The Transdanubian Hills were built of fluvial, Eolian material, and Palaeo- and Mesozoic marine sediments. During the Pleistocene, streams and rivers developed, and loess was deposited onto the surface, or intensive blown sand movement started on the dry sandy alluvial fans. The main soil types of the hills are brown forest soils with clay illuviation, and its subtype in the sandy areas. The soil is mostly covered by loess and sand^27^. This study is focused on sandy areas only.

### Studied habitats

We studied three endemic Pannonian sandy habitat types: (i) open sand steppes, (ii) closed sand steppes, and (iii) poplar-juniper sand dune forests and thickets, which are integrated into the European Union Natura 2000 network (92/43/EEC, I. Appendix: 6260, 2340, 91N0). Their total actual area is around 41.700 ha. The species composition of the studied habitats overlaps substantially (see Supplementary Data 2 based on Bölöni et al.^40^, Csecserits et al.^59^, Borhidi^64^, Horváth et al.^65^, Király et al.^66^): the grasslands are dominated by *Festuca* spp. and *Stipa* spp., the dune forests are dominated by *Juniperus communis* and *Populus* spp. At present, *Robinia pseudoacacia* plantations pose the main threat, destroying the habitats and opening the ground for further alien colonization^43^. Other threats include habitat loss, degradation, and fragmentation due to intensive agriculture and forestry, overgrazing, and wildfires^28,35,40,67^.

### Data sources

The Hungarian Habitat Mapping Database (MÉTA^68–70^) was used to study the regeneration capacity of the three sandy habitats. The MÉTA database is a result of a grid-based vegetation mapping of the whole territory of the country (2834 quadrats of 5.5 × 6.5 km rectangular landscape units), including about 200 mappers and more than 7000 days on the field. The occurrence of (semi-)natural habitats and habitat quality attributes (e.g., regeneration capacity, naturalness, main threats) were estimated based on field surveys.

In order to standardize the methodology, participants underwent three days of mandatory field training, located regenerating stands of all (semi-)natural habitat types, and used pre-printed datasheets to sort them into categories of regeneration capacity (Mapping Guide^68^ and Habitat Guide^71^). The Mapping Guide defines the terms and specifies the rules for filling out the datasheet. The Habitat Guide precisely describes the habitat types (habitat definition, site conditions, characteristic species, vegetation context, etc.). A large set of standard field examples for each habitat and for each regeneration category was developed to help the estimation of regeneration potential. The total number of examples given was 678 for regeneration. The following attributes were considered in relation to regeneration: the condition of the stand (e.g., species richness, regeneration ability of target species, the competitive ability of other species), abiotic site conditions (e.g., water supply, soil-nutrient balance, erosion), conditions of the landscape (e.g., disturbance level of the landscape, propagule sources, mobility of species related to their distance to the propagule source), type of land use (e.g., forest management, pasture)^69^.

Three types of locations were chosen to represent three types of regeneration capacity at the quadrat level. These included: (i) On-spot regeneration capacity is the capability of an existing stand to return to its natural state after a possible partial degradation in the future, i.e., the capacity of the habitat to reach the most natural state in the current landscape after degradation. The potential for regeneration was assessed based on local species richness and species composition, patch size, vegetation pattern, land use, neighboring habitat types, and the abundance of weeds. (ii) Regeneration capacity on a neighboring spot means the capability of the on-spot habitat to spread to immediately adjacent sites and restore itself by replacing the adjacent habitat without human intervention (e.g., if a meadow dries out due to the lowering of the groundwater table, it can be colonized by the species of a neighboring grassland). During the assessment, the main factors for consideration were the state of the local stands (including the colonization ability of species) and the state of the local landscape (disturbance level, barriers hindering the colonization, the site conditions of the adjacent habitats). (iii) Old-field regeneration capacity is the capability of the habitat to (re-)colonize abandoned open areas nearby the patch or even farther away within the studied landscape quadrat. In our case, open areas were abandoned arable fields. The regeneration capacity was estimated based on the ability of the local habitat type to colonize other areas and the availability of old fields with suitable site conditions in the landscape. The potential of each regeneration capacity type, from good to low (and impossible in the absence of suitable sites) were assessed on a four-point ordinal scale (Table 1).

**Table 1:** Scale for estimating regeneration capacity (based on Seregélyes et al.^18^).

Types of regeneration capacity	Description
good (4)	The regeneration capacity is considered good if there are adequate sources of species and suitable sites for regeneration within the landscape, which is likely to result in similar (semi-)natural habitats.
moderate (3)	If the landscape has some potential for regeneration, but it is either too slow or does not reach the natural state (e.g., remains dominated by weeds and indifferent species), then the regeneration capacity is considered moderate.
low (2)	In case the regeneration begins, some dominant species can colonize, but the process does not result in the original habitat type due to the lack of propagules or the rapid spread of invasive species (e.g., a large stand of weeds, the vegetation of weedy bushes), the regeneration capacity is low.
none (1)	In some landscapes, there is a lack of suitable spaces for colonization; therefore regeneration is impossible.

We included three groups of environmental predictors in our analysis (a detailed summary of proxies and the methods for aggregating environmental predictors at the quadrat level are given in Table 2) that potentially influence the regeneration capacity of sandy habitats in the studied quadrats: (i) proxies for landscape naturalness, (ii) landscape composition, and (iii) abiotic factors (Fig. 3a). We used the following proxies for landscape naturalness based on the MÉTA database: area; habitat naturalness; and the previous two approaches combined in the Natural Capital Index (NCI): NCI of sandy habitats and NCI of all the habitats; and invasion.

**Table 2:** A description of the environmental predictors and the data sources from which they were calculated.

Type of environmental predictor	Environmental predictor	Description	Data source	Level	Estimation/calculation method	Aggregation method to quadrat level	Range of values within quadrats	Unit
Proxy for landscape naturalness	Area	The quantity of (semi-)natural habitats, which means the total local extent of habitats	MÉTA	Hexagon	Expert judgment of % within 35 ha hexagon	Sum area of hexagons	0.03–819.36	ha
	Habitat naturalness	The estimated quality of the habitat	MÉTA	Hexagon	Expert judgment on 5-grade scale from totally degraded habitats to habitats with a high number of specialist and rare species^88^	Weighted average of naturalness where the weight was the extent of sandy habitats within a quadrat	1.99–5	non-unit scale
	NCI of sandy habitats	Natural capital index of sandy habitat	MÉTA	Hexagon	Based on ecosystem quality (naturalness) and ecosystem quantity (area)^89^	Average of NCI values of sandy habitats	0–11.63	%
	NCI of all habitats	Natural capital index of all habitats	MÉTA	Hexagon	Based on ecosystem quality (naturalness) and ecosystem quantity (area)^89^	Average of NCI values of all habitats	0–22.76	%
	Invasion	The extent of invaded area	MÉTA	Hexagon	Expert judgment of % within 35 ha hexagon	Sum area of hexagons	0–521.5	ha
Landscape composition	Artificial surfaces	Area of discontinuous urban fabric, industrial and commercial units, roads and networks, associated land, airports, mineral extraction sites, dump sites, construction sites, green urban areas, sport and leisure facilities	CLC 2006	CLC polygon	Mapping^72^	Sum area of CLC polygons	0–262.14	ha
	Agricultural areas	Area of non-irrigated arable land, vineyards, fruit trees and berry plantations, complex cultivation patterns, land principally occupied by agriculture with significant areas of natural vegetation	CLC 2006	CLC polygon	Mapping^72^	Sum area of CLC polygons	0.04–345.22	ha
	Forests	Area of broad-leaved forests, coniferous forests, mixed forests, transitional woodland scrubs	CLC 2006	CLC polygon	Mapping^72^	Sum area of CLC polygons	2.83–339.48	ha
	Grasslands	Area of natural grasslands, pastures, rare vegetation	CLC 2006	CLC polygon	Mapping^72^	Sum area of CLC polygons	0–221.41	ha
	Wetlands	Area of inland marshes, peat bogs	CLC 2006	CLC polygon	Mapping^72^	Sum area of CLC polygons	1.09–76.18	ha
	Water bodies	Area of water courses, water bodies	CLC 2006	CLC polygon	Mapping^72^	Sum of area of CLC polygons	0–168.89	ha
Abiotic factor	Soil	Maximum sand fraction ratio in the upper 0-30 cm soil layer	DOSoReMI; Somodi et al., 2017	Hexagon	Downscaling	Average of soil data	9.81–97.31	%
	Groundwater	Mean level of groundwater	DOSoReMI; Somodi et al., 2017	Hexagon	Downscaling	Avarage of groundwater data	1.48–6.47	m
	Topographic variation	Standard deviation of Topographic Position Index (TPI)	USGS; Somodi et al., 2017	Hexagon	Focal statistics	Average of topographic variation	74.67–305.14	m
	Temperature	Seasonality of thirty-year average temperature	CarpatClim-Hu; Somodi et al., 2017	Hexagon	Downscaling	Average of temperature data	737.32– 810.42	0.01°C
	Precipitation	Seasonality of thirty-year average precipitation	CarpatClim-Hu; Somodi et al., 2017	Hexagon	Downscaling	Average of precipitation data	0.18–0.33	Dimensionless ratio

To calculate the landscape context, we used the European CORINE Land Cover 2006 database^72^, which is the closest in time to the Hungarian MÉTA survey. The minimum mapping unit was 25 ha for habitat patches and 100 m width for linear landscape elements^73^. We merged the CLC classes into six categories according to land cover types: artificial surfaces; agricultural areas; forests; grasslands; wetlands and water bodies. The area of all CLC classes within the studied quadrats was calculated using ArcGIS 10.2 software^74^.

In addition, we selected abiotic factors that were good predictors for sandy vegetation nationwide in multiple potential natural vegetation models (MPNV^39^). These were as follows: soil properties; groundwater; topographic variation; temperature seasonality, and precipitation seasonality. Soil properties were obtained from the DOSoReMI.hu soil database^75^. Topographic Position Index (TPI) was calculated with a 3 × 3 focal matrix with the “raster” package^76^ of the R statistical software^77^. Elevation data for the TPI calculation were acquired from the SRTM digital terrain model^78^ that has 90 m horizontal and ca. 16 m vertical resolution^79^. Raw climate data were obtained from the CarpatClim-Hu database^80^ for the 1977–2006 period in daily temporal resolution and 0.1° (~10 km) horizontal resolution. All abiotic predictors were downscaled to the hexagon level and aggregated by Somodi et al.^30^.

### Statistics and reproducibility

First, we selected those mesoregions in Hungary where the number of sandy quadrats reaches a minimum of 30 (Mezőföld *n* = 36, Bácskai-síkvidék *n* = 50, Belső-Somogy *n* = 51, Dunamenti-síkság *n* = 81, Nyírség 125, Duna-Tisza közi síkság *n* = 294). We calculated the regeneration trajectories for each habitat type together. For this, we placed the three different locations (spot, neighboring spots, old fields) in order of distance on the x-axis and plotted the regenerative capacity values on the y-axis for each sampled quadrat. The regenerative capacity can take four different values (from good to low, plus lack of suitable spaces) at each location. The three location values together make up a spatial trajectory for a quadrate (at open sand grasslands *n* = 223, closed sand grasslands *n* = 363, and juniper-poplar stands *n* = 51) and the quadrates together for a mesoregion make up the spatial trajectory series (the detailed exact values for each habitat type and mesoregion are given in Supplementary Data 1). To detect differences between the mesoregions, we used the one-way ANOVA statistical method with Tukey’s honest post hoc significance test. The dependent variables were the spatial trajectory series and the fixed factors were the mesoregions. The mean difference was considered significant at the 0.05 probability level. The trajectory analysis was performed and the graphs were constructed using the IBM SPSS Statistics 17.0 software.

In a second step, the relationship between spatial regeneration trajectories and environmental predictors was analysed together for all mesoregions, but separately for the three sandy habitat types using the “randomForest” package^81^ in R statistical software^77^. Random Forest (RF) is an ensemble of Classification and Regression Trees trained on datasets of the same size as a training set, created from a random resampling on the training set itself. Once a tree is constructed, training sets, which do not include any particular record from the original dataset are used as a test set^82,83^. The advantage of tree-structured models is that the predictors are handled separately; therefore it is free from the problems caused by multicollinearity, so correlation analysis is not required^84^. To increase the accuracy of the RF model, we grouped the trajectory series into four major types: (1) flat, (2) decreasing, (3) V-shape, and (4) increasing trajectories. The number of randomly selected variables was three at each split. The RF models were trained with 3000 trees on environmental predictor datasets, and the feature importance ranking was extracted. The model’s performance was evaluated with the estimation of classification accuracy. The importance values are given in percentages.

The spatial autocorrelation of model residuals according to Moran’s I and its significance were studied with packages “sf”^85^ and “spdep”^86,87^ in the R statistical software^77^. Finally, we defined potential restoration interventions for the different regeneration capacity types locally and for each type of spatial regeneration trajectory.

### Reporting summary

Further information on research design is available in the Nature Research Reporting Summary linked to this article.

## Supplementary information

Supplementary Information

Description of Additional Supplementary Files

Supplementary Data 1

Supplementary Data 2

Reporting Summary

## Data Availability

The authors declare that the data supporting the findings of this study are available within the article, and its Supplementary information files. Any other relevant data are available from the corresponding author upon reasonable request.
