# Supplementary material for: Estimates of regeneration potential in the Pannonian sand region help prioritize ecological restoration interventions
Source: Commun Biol. 2022 Oct 27;5:1136. doi: 10.1038/s42003-022-04047-8 (PMC9613635; doi:10.1038/s42003-022-04047-8)
Supplement: Supplementary file 1 — Supplementary Information [file 42003_2022_4047_MOESM1_ESM.pdf]

Supplementary information

Estimates of regeneration potential in the Pannonian sand region help prioritize ecological restoration interventions

Edina Csákvári[1,*], Zsolt Molnár[1], Melinda Halassy[1]

[1]ELKH Centre for Ecological Research, Institute of Ecology and Botany, Alkotmány u. 2-4, 2163 Vácrátót, Hungary

[*]Corresponding author at: ELKH Centre for Ecological Research, Institute of Ecology and Botany, Alkotmány u. 2-4, 2163 Vácrátót, Hungary. Phone number: +421 944 909 371, e-mail address: csakvari.edina@ecolres.hu (E. Csákvári).

Supplementary Tables

Table S1. Comparison of trajectory series in open sand steppes by one-way Anova.

	Sum of Squares	df	Mean Square	F	Sig.
Between Groups	2114,313	5	422,863	3,876	,002
Within Groups	23676,620	217	109,109		
Total	25790,933	222			

Table S2. Comparison of trajectory series in closed sand steppes by one-way Anova.

	Sum of Squares	df	Mean Square	F	Sig.
Between Groups	3493,245	5	698,649	7,589	,000
Within Groups	32866,656	357	92,063		
Total	36359,901	362			

Table S3. Comparison of trajectory series in closed sand steppes by one-way Anova.

	Sum of Squares	df	Mean Square	F	Sig.
Between Groups	959,468	2	479,734	13,409	,000
Within Groups	1717,238	48	35,776		
Total	2676,706	50			

Table S4. Multiple comparisons and Tukey's post hoc significance test between mesoregions in trajectory series in open sand steppes.

(I) Mesoregion	(J) Mesoregion	Mean Difference (I-J)	Std. Error	Sig.	95% Confidence Interval Lower Bound	Upper Bound
Belső-Somogy	Bácskai-síkvidék	1,705	4,107	,998	-10,10	13,51
	Dunamenti-síkság	-2,097	4,083	,996	-13,84	9,64
	Duna-Tisza közi síkság	-1,196	3,635	,999	-11,65	9,26
	Mezőföld	-1,984	5,264	,999	-17,12	13,15
	Nyírség	5,694	3,734	,648	-5,04	16,43
Bácskai-síkvidék	Belső-Somogy	-1,705	4,107	,998	-13,51	10,10
	Dunamenti-síkság	-3,803	3,048	,813	-12,57	4,96
	Duna-Tisza közi síkság	-2,901	2,416	,836	-9,85	4,04
	Mezőföld	-3,689	4,509	,964	-16,65	9,28
	Nyírség	3,989	2,562	,628	-3,38	11,35
Dunamenti-síkság	Belső-Somogy	2,097	4,083	,996	-9,64	13,84
	Bácskai-síkvidék	3,803	3,048	,813	-4,96	12,57
	Duna-Tisza közi síkság	,902	2,374	,999	-5,93	7,73
	Mezőföld	,113	4,487	1,000	-12,79	13,01
	Nyírség	**7,792***	2,523	,027	,54	15,05
Duna-Tisza közi síkság	Belső-Somogy	1,196	3,635	,999	-9,26	11,65
	Bácskai-síkvidék	2,901	2,416	,836	-4,04	9,85
	Dunamenti-síkság	-,902	2,374	,999	-7,73	5,93
	Mezőföld	-,789	4,084	1,000	-12,53	10,95
	Nyírség	**6,890***	1,706	,001	1,99	11,79
Mezőföld	Belső-Somogy	1,984	5,264	,999	-13,15	17,12
	Bácskai-síkvidék	3,689	4,509	,964	-9,28	16,65
	Dunamenti-síkság	-,113	4,487	1,000	-13,01	12,79
	Duna-Tisza közi síkság	,789	4,084	1,000	-10,95	12,53
	Nyírség	7,679	4,172	,442	-4,32	19,67
Nyírség	Belső-Somogy	-5,694	3,734	,648	-16,43	5,04
	Bácskai-síkvidék	-3,989	2,562	,628	-11,35	3,38
	Dunamenti-síkság	**-7,792***	2,523	,027	-15,05	-,54
	Duna-Tisza közi síkság	**-6,890***	1,706	,001	-11,79	-1,99

	Mezőföld	-7,679	4,172	,442	-19,67	4,32

*. The mean difference is significant at the 0.05 level.

Table S5. Multiple comparisons and Tukey's post hoc significance test between mesoregions in trajectory series in closed sand steppes.

(I) Mesoregion	(J) Mesoregion	Mean Difference (I-J)	Std. Error	Sig.	95% Confidence Interval	
					Lower Bound	Upper Bound
Belső-Somogy	Bácskai-síkvidék	2,948	2,405	,824	-3,94	9,84
	Dunamenti-síkság	1,956	2,022	,928	-3,84	7,75
	Duna-Tisza közi síkság	**7,743***	1,690	,000	2,90	12,58
	Mezőföld	-,003	2,328	1,000	-6,67	6,67
	Nyírség	**5,892***	1,908	,026	,43	11,36
Bácskai-síkvidék	Belső-Somogy	-2,948	2,405	,824	-9,84	3,94
	Dunamenti-síkság	-,992	2,320	,998	-7,64	5,65
	Duna-Tisza közi síkság	4,794	2,037	,176	-1,04	10,63
	Mezőföld	-2,951	2,591	,865	-10,38	4,47
	Nyírség	2,944	2,222	,771	-3,42	9,31
Dunamenti-síkság	Belső-Somogy	-1,956	2,022	,928	-7,75	3,84
	Bácskai-síkvidék	,992	2,320	,998	-5,65	7,64
	Duna-Tisza közi síkság	**5,787***	1,566	,003	1,30	10,27
	Mezőföld	-1,959	2,240	,952	-8,38	4,46
	Nyírség	3,936	1,799	,246	-1,22	9,09
Duna-Tisza közi síkság	Belső-Somogy	**-7,743***	1,690	,000	-12,58	-2,90
	Bácskai-síkvidék	-4,794	2,037	,176	-10,63	1,04
	Dunamenti-síkság	**-5,787***	1,566	,003	-10,27	-1,30
	Mezőföld	**-7,745***	1,945	,001	-13,32	-2,17
	Nyírség	-1,850	1,416	,781	-5,91	2,21
Mezőföld	Belső-Somogy	,003	2,328	1,000	-6,67	6,67
	Bácskai-síkvidék	2,951	2,591	,865	-4,47	10,38
	Dunamenti-síkság	1,959	2,240	,952	-4,46	8,38
	Duna-Tisza közi síkság	**7,745***	1,945	,001	2,17	13,32
	Nyírség	5,895	2,138	,067	-,23	12,02
Nyírség	Belső-Somogy	**-5,892***	1,908	,026	-11,36	-,43

	Bácskai-síkvidék	-2,944	2,222	,771	-9,31	3,42
	Dunamenti-síkság	-3,936	1,799	,246	-9,09	1,22
	Duna-Tisza közi síkság	1,850	1,416	,781	-2,21	5,91
	Mezőföld	-5,895	2,138	,067	-12,02	,23

*. The mean difference is significant at the 0.05 level.

Table S6. Multiple comparisons and Tukey's post hoc significance test between mesoregions in trajectory series in poplar-juniper stands.

(I) Mesoregion	(J) Mesoregion	Mean Difference (I-J)	Std. Error	Sig.	95% Confidence Interval	
					Lower Bound	Upper Bound
Bácskai-síkvidék	Dunamenti-síkság	**-14,333***	4,229	,004	-24,56	-4,10
	Duna-Tisza közi síkság	-,952	3,575	,962	-9,60	7,69
Dunamenti-síkság	Bácskai-síkvidék	**14,333***	4,229	,004	4,10	24,56
	Duna-Tisza közi síkság	**13,381***	2,610	,000	7,07	19,69
Duna-Tisza közi síkság	Bácskai-síkvidék	,952	3,575	,962	-7,69	9,60
	Dunamenti-síkság	**-13,381***	2,610	,000	-19,69	-7,07

*. The mean difference is significant at the 0.05 level.

Supplementary Figures

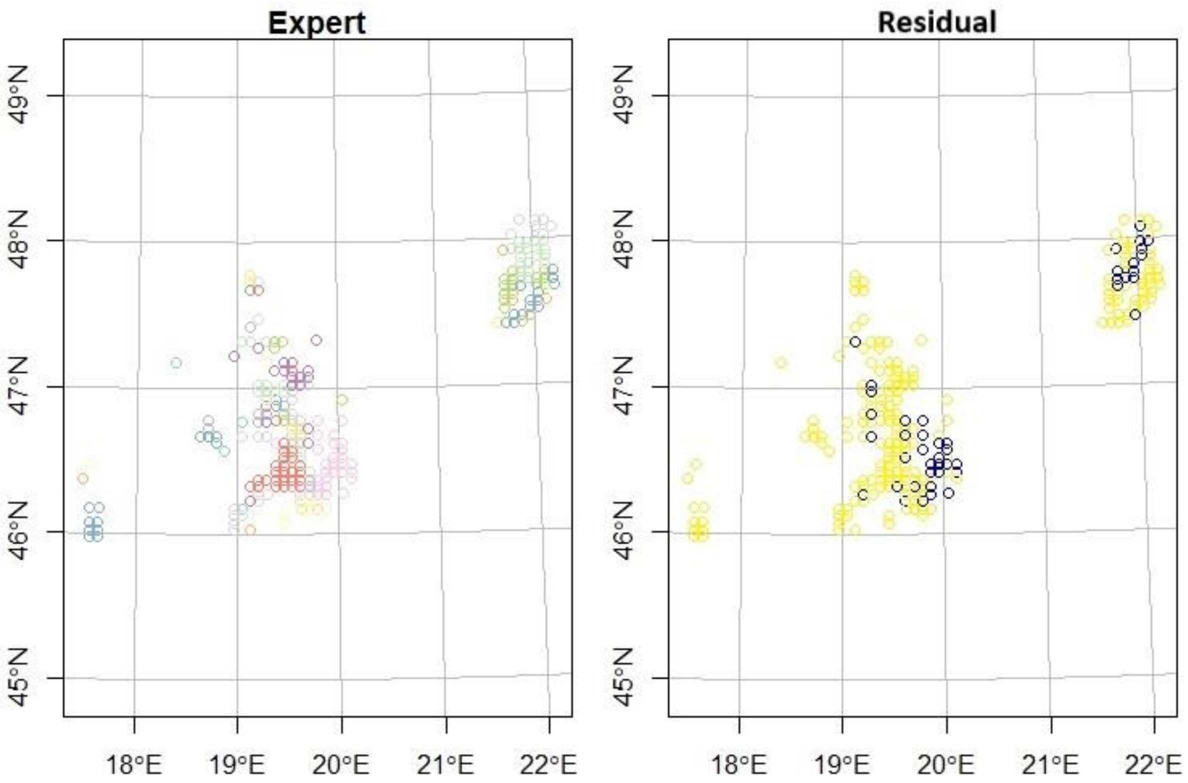

Figure S1. Spatial autocorrelation of open sand steppes (Moran's I = 0.13; $p < 0.01$) in the six Hungarian mesoregions.

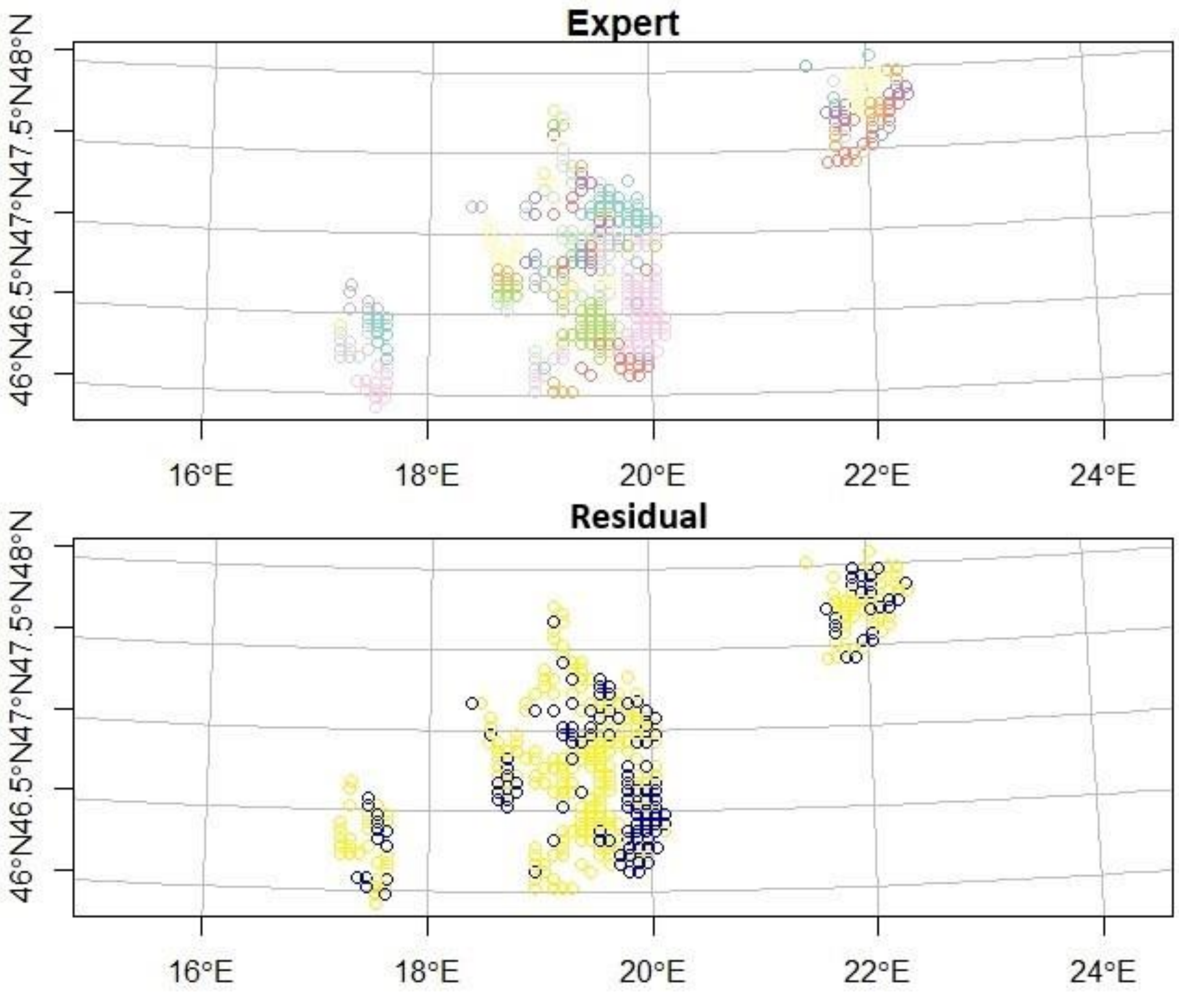

Figure S2. Spatial autocorrelation of closed sand steppes (Moran's I = 0.22; $p < 0.0001$) in the six Hungarian mesoregions.

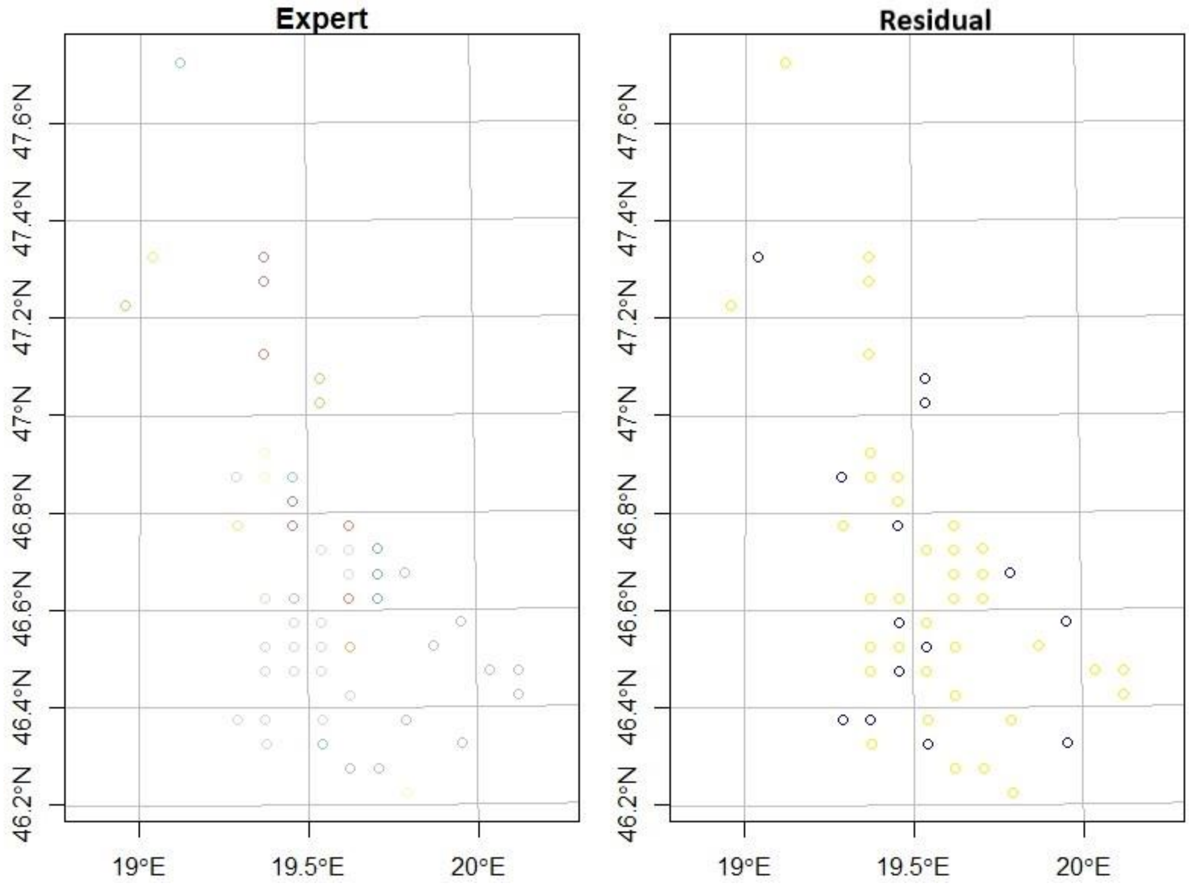

Figure S3. Spatial autocorrelation of poplar-juniper stands (Moran's I = 0.05; p = 0.29) in the three Hungarian mesoregions.

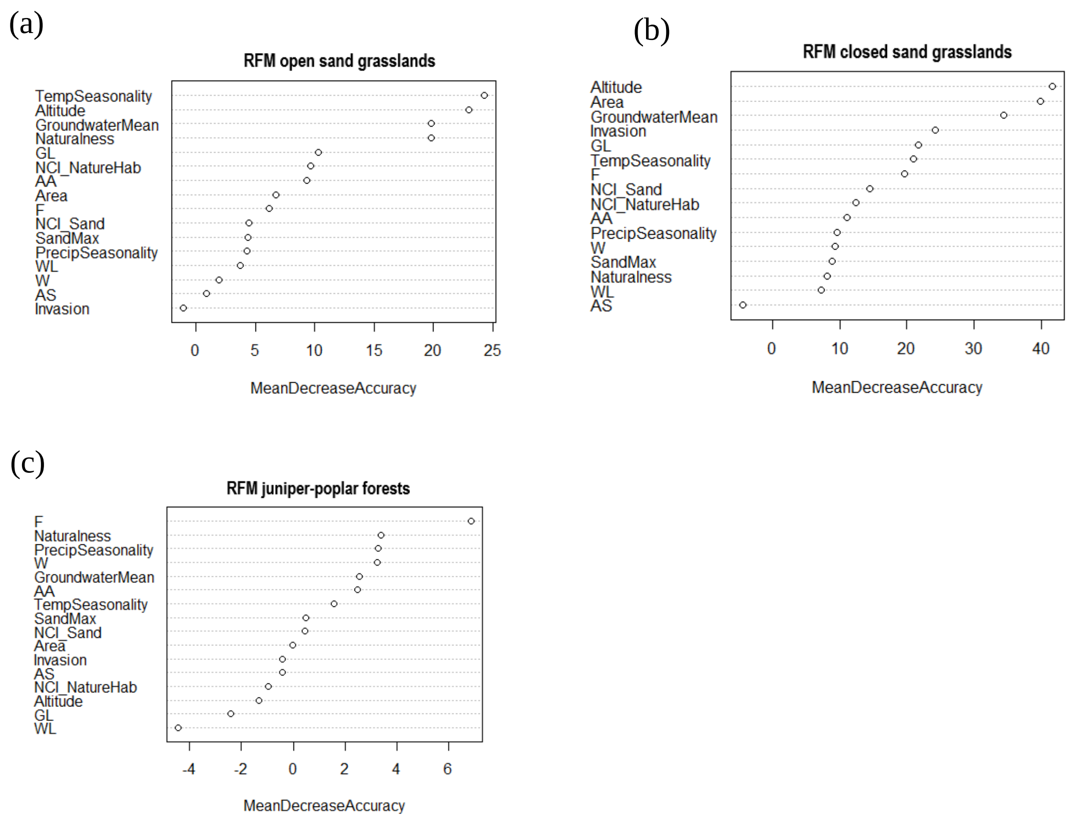

Figure S4. The mean decrease accuracy of environmental predictors determining the spatial regeneration trajectories in a) open and b) closed sand grasslands, and c) juniper-poplar stands within the studied mesoregions.
