# Supplementary material for: Estimates of regeneration potential in the Pannonian sand region help prioritize ecological restoration interventions
Source: Commun Biol. 2022 Oct 27;5:1136. doi: 10.1038/s42003-022-04047-8 (PMC9613635; doi:10.1038/s42003-022-04047-8)
Supplement: Supplementary file 2 — Description of Additional Supplementary Files [file 42003_2022_4047_MOESM2_ESM.pdf]

Description of Additional Supplementary Files

File name: Supplementary Data 1

Description: The number of spatial regeneration trajectories in each habitat type and mesoregion. Abbreviations: G1: open sand steppes, H5b: closed sand steppes, M5: poplar-juniper sand dune forests and thickets (Bölöni et. al, 2011). BESO: Belső-Somogy, BS: Bácskai-síkvidék, DS: Dunamenti-síkság, DTS: Duna-Tisza közi síkság, M: Mezőföld, Ny: Nyírség. 4: good, 3: moderate, 2: low, 1: impossible potential for regeneration.

File name: Supplementary Data 2

Description: Categorization of species according to their occurrence in the three sandy habitat types, social behaviour types and naturalness values. Abbreviations: ++ = frequent, + = present, (+) = disappeared, extinct, very rare or very sporadic, - = absent, C = competitor, Cu = unique competitor, S = specialist, Sr = rare specialist, Su = unique specialist, G = generalist, NP = natural pioneer, DT = disturbance tolerant, W = native weed, I = introduced alien species, RC = ruderal competitor, AC = alien competitor. Naturalness value scale: from -3 (threatful to nature) to +9 (value to nature).
